# 510 Elevated Concentrations of IL-10 Secreted from Adipose-Derived Stem Cells of Burn Patients with Higher BMIs

**DOI:** 10.1093/jbcr/iraf019.139

**Published:** 2025-04-01

**Authors:** Kaitlyn Andre, Paige Deville, Abdul-Razak Masoud, Dhanushka Vitharana, Ada Ozcan, Jeffrey Carter, Herbert Phelan, Jonathan Schoen, Victoria Miles, Alison Smith

**Affiliations:** Louisiana State University Health Sciences Center - New Orleans; Louisiana State University Health Sciences Center - New Orleans; Louisiana State University Health Sciences Center - New Orleans; Louisiana State University Health Sciences Center - New Orleans; Louisiana State University Health Sciences Center - New Orleans; Burn Center at University Medical Center; Burn Center at University Medical Center; Louisiana State University Health Sciences Center - New Orleans; University Medical Center New Orleans; Louisiana State University Health Sciences Center - New Orleans

## Abstract

**Introduction:**

Human adipose tissue has known metabolic and endocrine activity which drives the immune response and healing process following injury. In obese patients, adipose tissue remodeling can cause cell death and mechanical stress which contribute to chronic inflammation. As cytokines play a key role in regulating cellular metabolism, we hypothesized that the ADSCs of patients with higher BMIs would display significantly different cytokine profiles than those with lower BMIs.

**Methods:**

Following IRB approval, subcutaneous adipose tissue was collected from adult burn patients at the site of injury during the index operation. ADSCs were isolated with flow cytometry. Each sample was grown for 24 hours using standard cell culture techniques. Supernatant was extracted and tested using a 10-analyte multiplex assay targeting IFN-𝛾, IL-1β, IL-4, IL-6, IL-10, IL-13, IL-17A, TGF-α, TNF-α, FGF-2, MCP-1, and VEGF. Patients were stratified based on BMIs either higher or lower than 25 kg/m^2, and a 2-tailed t-test was performed.

**Results:**

The average BMI of the 27 patients studied was 26.64±6.19kg/m^2. Over half of the patients (52%, n=14/27) had a BMI >25 kg/m^2. Within these patients, levels of IL-10 were significantly higher (p=0.02) than the 13 patients with a BMI of < 25 kg/m^2. No significant differences in levels of the remaining cytokines were identified between the two groups (p>0.05).

**Conclusions:**

This study furthers our understanding of the cytokine levels in ADSCs of burn patients with higher BMIs. As IL-10 has known anti-inflammatory properties, increased levels may limit the host cell immune response, increasing susceptibility to infection. Further studies are needed to determine the impact of this finding on wound healing in this high-risk population.

**Applicability of Research to Practice:**

The cytokine profiles of ADSCs in burn patients may contribute to the development of targeted therapeutics for burn wounds. These treatments could include stem cell-derived biological therapeutics that may promote faster wound healing and decrease the risk of infection.

**Funding for the Study:**

N/A

